# Investigation of Releasing Chamomile Essential Oil from Inserts with Cellulose Agar and Microcrystalline Cellulose Agar Films Used in Biotextronics Systems for Lower Urinary Tract Inflammation Treatment

**DOI:** 10.3390/ma17164119

**Published:** 2024-08-20

**Authors:** Emilia Frydrysiak, Krzysztof Śmigielski, Alina Kunicka-Styczyńska, Michał Frydrysiak

**Affiliations:** 1 Institute of Natural Products and Cosmetics, Faculty of Biotechnology and Food Sciences, Lodz University of Technology, ul. Stefanowskiego 2/22, 90-537 Lodz, Poland; 2 Department of Environmental Biotechnology, Faculty of Biotechnology and Food Sciences, Lodz University of Technology, ul. Wólczańska 171/173, 90-530 Lodz, Poland; krzysztof.smigielski@p.lodz.pl; 3 Department of Sugar Industry and Food Safety Management, Faculty of Biotechnology and Food Sciences, Lodz University of Technology, ul. Wólczańska 171/173, 90-530 Lodz, Poland; alina.kunicka@p.lodz.pl; 4 Institute of Materials Science of Textiles and Polymer Composites, Faculty of Material Technologies and Textile Design, Lodz University of Technology, ul. Żeromskiego 116, 90-543 Lodz, Poland; michal.frydrysiak@p.lodz.pl

**Keywords:** chamomile essential oil, lower urinary tract inflammation, cellulose, microcrystalline cellulose, agar film, insert, SPME, GC–MS, biotextronics

## Abstract

Lower urinary tract inflammation is a very common problem which occurs particularly in women. That is why the idea of a biotextronics system for preventive and supportive treatment came to be. The system is a kind of a therapeutic clothing in the form of underwear integrated with a four-layer pantiliner with biological active compounds (from chamomile essential oil) immobilized on the insert with a cellulose agar or microcrystalline cellulose agar film. In this research, the outer part of the insert was investigated for its ability to release compounds with antibacterial and anti-inflammatory activity under the temperature of the treatment (40 °C). The research was conducted on the day of the insert preparation (day 0) and also after 7, 14, 28, and 56 days to test the ability of the insert to be stored without changing its properties. The results showed that even after 56 days of storage, there are compounds released that are known to have antibacterial activity, such as *α*-bisabolol. The system requires further tests involving bacteria; however, chamomile essential oil seems to be good substrate for biotextronics systems for preventive and supportive treatment of lower urinary tract inflammations.

## 1. Introduction

Lower urinary tract inflammation is a very common problem which occurs particularly in women. It occurs 50 times more often in women than in men [1]. It is estimated that every second woman suffers from a urinary tract infection at least once in her life [2]. This is due to having a short urethra and the small distance between its opening and the vaginal opening and anus. This distance is important because the most common cause of lower urinary tract infections is infection with *Escherichia coli*, which lives in the large intestine [3,4].

There are four possible routes for microorganisms entering the urinary tract: lymphatic, blood-borne, through continuity, and ascending. Infection with microorganisms through the blood-borne route is rare and most often affects newborns and people with reduced immunity. This route is mainly used by staphylococci, streptococci, *Mycobacterium*, and yeasts of the *Candida* genus, but rarely Gram-negative rods. The presence of entero-urinary fistulas causes the penetration of microorganisms through continuity. In this case, microorganisms pass into the urinary tract directly from the digestive tract [5,6]. The most common cause of lower urinary tract inflammation, both out-of-hospital and in-hospital, is bacterial infection through the ascending route [6,7]. Extra hospital infections in this way are mainly related to the specific anatomical structure of the female body and a lack of proper hygienie. Hospital infections are mainly caused by urinary retention, previous urological procedures, catheterization, and a lack of proper hygiene. Insertion of a catheter into the bladder increases the probability of infection by 10%, while with each additional day of keeping the catheter in the urinary tract, the risk of infection increases by 3–10% [6]. The list of microorganisms causing lower urinary tract inflammation is presented below (Table 1).

To treat lower urinary tract inflammations, antibiotics are usually used, but their frequent use can lead to vaginal and intestinal dysbiosis, as well as antibiotic resistance of microorganisms. Therefore, preventive measures are desirable to prevent urinary tract infections. Numerous clinical studies indicate that substances of natural origin can provide effective prophylaxis in the case of recurrent infections. The most effective are, among others, cranberries, berberine, essential oils, probiotics, and vitamins [10,11].

Home remedies for relieving symptoms that occur during inflammation of the urethra and bladder include staying warm [6,7]. In the case of relieving pain located in the lower abdomen, warm baths lasting no longer than 5–10 min may be helpful [7]. Essential oils with anti-inflammatory and antiseptic properties can be used for baths, including chamomile or sage essential oils [12].

In previous research, the essential oil (EO) from chamomile (*Matricaria chamomilla* L.) applied on viscose non-woven inserts has been investigated for its antibacterial activity in the gas phase against bacteria that cause lower urinary tract inflammations: *Escherichia coli*, *Staphylococcus saprophyticus*, *Staphylococcus epidermidis*, *Enterococcus faecalis*, and *Pseudomonas aeruginosa*. It was observed that the EO had various impacts on individual bacteria species. The activity of the chamomile essential oil was measured as a biomass loss. The best results were obtained against *S. saprophyticus* (38% of biomass loss) and *E. faecalis* (33% of biomass loss) in an EO concentration of 0.204 µL/cm^3^. At the concentrations of the essential oil in the atmosphere of 0.054 µL/cm^3^ and 0.106 µL/cm^3^, chamomile EO caused a loss of biomass for all tested bacterial strains. As indicated by the literature data, in addition to the tested microbial strains, chamomile essential oil is active against other bacteria associated with lower urinary tract infections, such as *Staphylococcus aureus* bacteria, causing mainly hospital infections, and *Proteus* bacteria, which are responsible for recurrent infections [1,6,7].

In addition to pharmacological treatment, a common way to combat lower urinary tract inflammation are steam baths. Some doctors recommend using them from several times a week to even two to three times a day in the initial phase of treatment, for 15–20 min. Various essential oils are often added to the water, including chamomile, which supports healing and relieves discomfort [12]. However, the use of steam baths is not very comfortable and does not allow mobility during its use.

**Figure 1:**
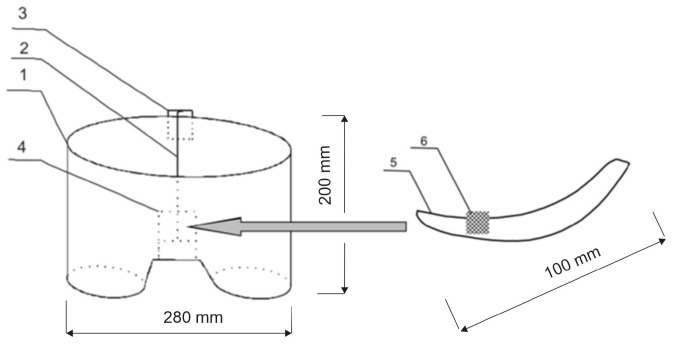
Schematic diagram of the biotextronics system: 1—textronics underwear; 2—textile signal line; 3—regulation system; 4—textronics heating insert; 5—outer (removable) insert; and 6—essential oils placed on the insert [1].

**Figure 2:**
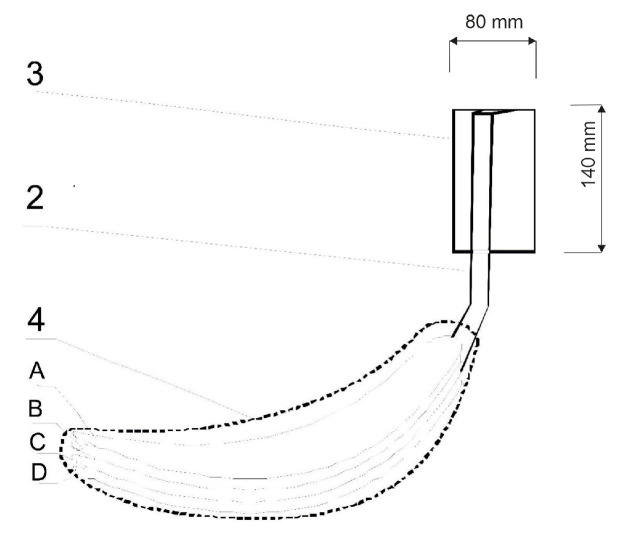
Diagram of the biotextronics insert: 4—heating insert (A—insert with applied essential oils; B and D—electrical insulating layers; C—system heating element); 2—textile signal line; and 3—battery [1].

That is why the idea of a biotextronics system (acronym BioTexPants) for preventive and supportive treatment came to be. The system is a kind of a therapeutic clothing in the form of underwear integrated with a four-layer pantiliner with biological active compounds (Figure 1 and Figure 2) [1]. Such a solution is a combination of cosmetotextiles with healing effects.

Cosmetotextiles are textiles with active compounds as cosmetic ingredients, which are transferred and have the ability to ensure an optimal exchange in order to generate beneficial effects regarding skin appearance. Through the use of cosmetotextiles, well-being and health can be improved where certain substances or even vitamins could increase skin health. Moreover, these textile materials establish the basis for the delivery system of biologically active substances in contact with the skin [13].

The biotextronics system for the prevention and supportive treatment of lower urinary tract infections consists of underwear (Figure 1(1)) with a permanently attached textronics insert (Figure 1(4)) on which a disposable, replaceable insert with applied essential oil is placed (Figure 1(6)). The outer insert has anti-inflammatory and antibacterial properties thanks to the applied essential oil, released from the insert under the influence of higher temperatures (40 °C, which is the temperature for therapy). The textronics (heating) insert consists of three layers B–D (Figure 2). Layers B and D are electrically insulating layers, with layer B characterized by good thermal conductivity properties. Layer C is made of electroconductive textile materials, and it is the heating element of the system, connected via a textile signal line (Figure 1(2)) to the regulation system (Figure 1(3)) that ensures maintaining the appropriate temperature during therapy. The textronics insert together with the replaceable insert (Figure 1(5) and Figure 2(A)) with applied essential oils form the biotextronics insert. Power to the biotextronics system can be supplied via a 5 V lithium-polymer battery [1].

The essential oil is applied on the insert with an agar film and cellulose or microcrystalline cellulose as carriers. Cellulose is the most abundant biopolymer with extensive sources in nature [14,15]. It is a natural long-chain polymer that plays an important role in the human food cycle, indirectly. This polymer has versatile uses in many industries such as veterinary foods, wood and paper, fibers and clothes, and the cosmetic and pharmaceutical industries as an excipient. Pure cellulose is available in different forms in the market with very different mechanical and pharmaceutical properties. The difference between various forms of cellulose is related to the shape, size, and degree of crystallinity of their particles (fibrous or agglomerated). Microcrystalline cellulose is the most known cellulose, which is extensively used in pharmaceutical industries. Microcrystalline cellulose grades are multifunctional pharmaceutical excipients which can be used as compressibility enhancers, binders in wet and dry granulation processes, thickeners and viscosity builders in liquid dosage forms, and free-flowing agents in solid dosage forms. The mechanical properties of microcrystalline cellulose grades are greatly influenced by their particle size and degree of crystallization [16].

In the previous research, there was an investigation of five essential oils from *Matricaria chamomilla* L., *Salvia officinalis* L., *Salvia lavandulaefolia* Vahl., and *Juniperus communis* L. *Thymus vulgaris* L., which were concluded to have anti-inflammatory and antibacterial activity. EOs were investigated to examine their antimicrobial activity against bacteria that cause lower urinary tract inflammations: *Escherichia coli, Staphylococcus saprophyticus, Staphylococcus epidermidis, Pseudomonas aeruginosa*, and *Enterococcus faecalis*. Chamomile essential oil was chosen to be used in a model outer insert with antibacterial activity [1].

Chamomile essential oil was obtained during classic steam distillation, lasting from 7 to 13 h. The repeated distillation allowed the oil extraction efficiency to be increased by 30%. The efficiency of the process was low and amounted to approximately 0.20–0.63%. The EO was characterized by a light blue color, which was caused by the terpenoid–chamazulene [17]. The main ingredients of chamomile EO include *α*-bisabolol (1–60%), chamazulene (2–25%), en-in-dicycloether (6%), *α*-bisabolol A oxide (2–60%), n-octanal (6.0%), *α*-bisabolol B oxide (3–50%), *α*-bisabolone A oxide (0.4–12%), 1,8-cineole (3.9%), (E)-*β*-farnesene (5–40%), *α*-terpineol (approx. 3%), germacrene D (3%), (E)-(Z)-spiroethers (4–18%), and polyacetylene derivatives [18,19,20]. It is *α*-bisabolol which is mainly responsible for the antibacterial properties; however, chamazulene also has antimicrobial activity against Gram-positive bacteria [21].

*α*-Bisabolol, one of the main ingredients of chamomile oil, has a relaxing effect on intestinal smooth muscles. It also has anti-inflammatory, antibacterial, antifungal, and antipyretic properties, and prevents the formation of ulcers. Chamazulene, produced in the oil during distillation, has anti-inflammatory, antiallergic, and antispasmodic properties. Other components of the oil have similar properties, as well as carminative properties due to apigenin, flavonoids, and luteolin. Apigenin also binds to GABA receptors (*γ*-aminobutyric acid-binding membrane receptors) and has a mild sedative effect. Spiroethers, *cis*- and *trans*-en-ino-dicycloethers, have spasmolytic, antifungal, and anti-inflammatory properties [19,21]. Coumarin umbelliferone is assessed as an antispasmodic, antibacterial, and antifungal substance. It is generally accepted that the biological activity of the oil is caused by its main components, i.e., *α*-bisabolol and its oxides and azulenes, including chamazulene [19].

Blue chamomile essential oil is used in the food and pharmaceutical industries, and also as a coloring agent in food. In the production of cosmetics, it is an active substance in soaps, bath liquids, shower gels, shampoos for light hair, body balms, cosmetic masks, soothing creams, and for hand and foot care [17,22]. It is used to treat many diseases, including sleep disorders, anxiety, as a digestive aid, and skin infections and inflammations, including eczema. It also supports wound healing, relieves infant colic, reduces pain during teething and irritation caused by using diapers [23]. It is used to treat malaria, flu, and colds [21]. Moreover, chamomile oil is characterized by anti-itching, decongesting, anti-allergic, and healing properties [24].

Chamomile essential oil is used in the form of inhalation, massages, or bathing in cases of anorexia. In the form of inhalation, it is also used by patients suffering from asthma to increase airway patency. In the case of athlete’s foot, the oil is applied directly to the skin. You can also add it to bath water. Massages with the addition of oil also bring relief to people with the so-called hallux. In the case of wounds and scratches, spraying the oil directly on the site of the cut stimulates the growth of new cells. Inhalations with the addition of oil relieve stress during hypertension. In the case of juvenile rheumatoid arthritis, osteoarthritis, Ménière’s disease, knee and back pain, as well as problems related to menstruation, chamomile oil used in the form of massages leads to a reduction in disease symptoms [22].

**Figure 3:**
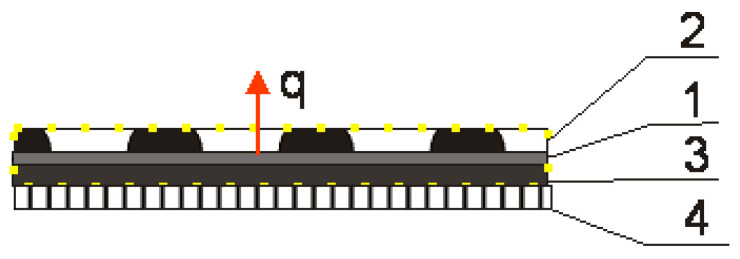
Diagram of the biotextronics system clothing package: 1—heating element of the textronics insert; 2—outer insulating layer with chamomile essential oil; 3—inner insulating layer; and 4—layer of underwear. q—heat flow; yellow dots area—area of the biotextronics insert [1].

The aim of this study was to determine the composition of chamomile essential oil and the above-surface phase of an insert material with biological active compounds from chamomile EO under a temperature of 40 °C, which is the temperature for therapy. The insert was made with non-woven viscose and the EO was dispersed with cellulose or microcrystalline cellulose as carriers in an agar-agar solution and applied on the insert as a film. Non-woven fabric was chosen due to its light weight, air permeability, and ease of processing [25]. Unlike conventional inserts, the BioTexPants outer insert is covered with an agar film. Natural biopolymers such as alginate, gelatin, chitosan, agar, guar gum, starch, etc., are considered to be favorable alternatives to non-biodegradable materials as conventional inserts, due to their excellent characteristics of film-forming properties, safety, non-toxicity, and low production cost. Agar possesses excellent film-forming ability and biocompatibility and is a kind of polysaccharide compound that can form gels without adding any coagulant aid. The shape of the agar film is stably maintained when presented in high-humidity surroundings and exhibit good heat seal performance [26]. The cellulose and microcrystalline cellulose was added with essential oil to the agar film as a carrier, and it was reported that microcrystalline cellulose was used as a carrier for hydrophobic substances in water and the essential oil is a hydrophobic product [27]. Essential oil is released from the pantiliner at a temperature of 40° from the textronics part of the underwear. The heat flow is presented in Figure 3:

The aim of the research was to prove that the compounds of chamomile essential oil may be released from the pantiliner and also that even during storage of the insert, compounds considered to be antibacterial and anti-inflammatory are released.

The above-surface phase compounds were investigated with the use of the microextraction to solid phase (SPME) method and identified with the use of gas chromatography–mass spectrometry (GC–MS). The research was conducted on the day of preparing the insert and after 7, 14, 28, and 56 days to determine the impact of the insert storage.

## 2. Materials and Methods

### 2.1. Materials

Materials used in the outer insert preparation are listed in Table 2.

Characteristics of chamomile EO are presented in Table 3.

### 2.2. Methods

#### 2.2.1. Gas Chromatography–Mass Spectrometry (GC–MS)

The quantitative and qualitative composition of the gas phase above the model inserts was determined using gas chromatography coupled with mass spectrometry (GC–MS). A coupled gas chromatograph was used with a Pegasus 4D mass spectrometer, with a TOF MS spectrometer (LECO, St. Joseph, MI, USA).

First-dimension column: Stabilwax-DA (Restek, Lisses, France) 30 m long, a 0.25 mm internal diameter, and a 0.25 µm stationary phase film thickness; second-dimension column: BPX-50 (SGE Analytical Science, Victoria, Australia) with a length of 2 m, an internal diameter of 0.1 mm, and a stationary phase film thickness of 0.1 µm. Carrier gas: helium, constant flow of 1 mL/min. First-dimension furnace temperature program: 50 °C (1 min), 4 °C/min to 245 °C (30 min); the temperature program of the second-dimension furnace shifted by +5 °C compared to the first-dimension furnace. Two-stage modulator was cooled to −80 °C. Modulation time was 8 s. Cold pulse time was 2.4 s. Hot pulse time was 1.6 s (+20 °C relative to the first-dimension furnace). SSL dispenser: temperature of 250 °C with a division of 1:30. Transfer line temperature: 280 °C. TOF mass spectrometer, detector voltage of 1600 V, ion source temperature of 200 °C, ionization energy of 70 eV, mass range of 33–350 amu, and scanning frequency of 150 spectra/s.

Compounds were identified using a chromatographic analysis of mass spectra, indices, and retention times, which were compared to Wiley, Adams, and Nist libraries.

The GC–MS was also used in the detection of compounds released in solid phase microextraction.

#### 2.2.2. Preparing the Model Inserts

The model inserts were prepared to fit into the SPME vial dimensions. Circles with diameters of 2.2 cm were cut out from the viscose and put into the vials. Agar was dissolved in hot water with constant stirring and cooled, and essential oil mixed with cellulose or microcrystalline cellulose was added to it. The concentration of the EO was constant and the concentrations of cellulose and microcrystalline cellulose were variable, equal to the EO weight quantity, double its amount, and triple its amount. Then, 3.2 mL of agar with cellulose and EO were put onto the inserts in vials. Such prepared inserts were model materials investigated for compounds in a gas phase above the inserts at a temperature of 40 °C, which is the temperature for therapy, on the day of their preparation (time 0) and also after 7, 14, 28, and 56 days of their storage.

#### 2.2.3. Solid Phase Microextraction (SPME)

Essential oil from chamomile was applied to the model insert and retained on it using an agar film with the addition of cellulose or microcrystalline cellulose, which constituted a model system. Volatile oil compounds released under the influence of an elevated temperature (40 °C) were determined during solid phase microextraction (SPME) and identified using gas chromatography coupled with mass spectrometry. Solid phase microextraction conditions were as follows:

- Temperature: 40 °C;
- Conditioning time: taq = 5 and 10 min;
- Extraction time: tex = 20 min;
- No mixing;
- Fiber: ternary DVB/CAR/PDMS (Sigma-Aldrich, USA).

#### 2.2.4. Software Tools

All the figures in the manuscript were created with the use of CorelDRAW Graphics Suite X3.

## 3. Results

**Figure 4:**
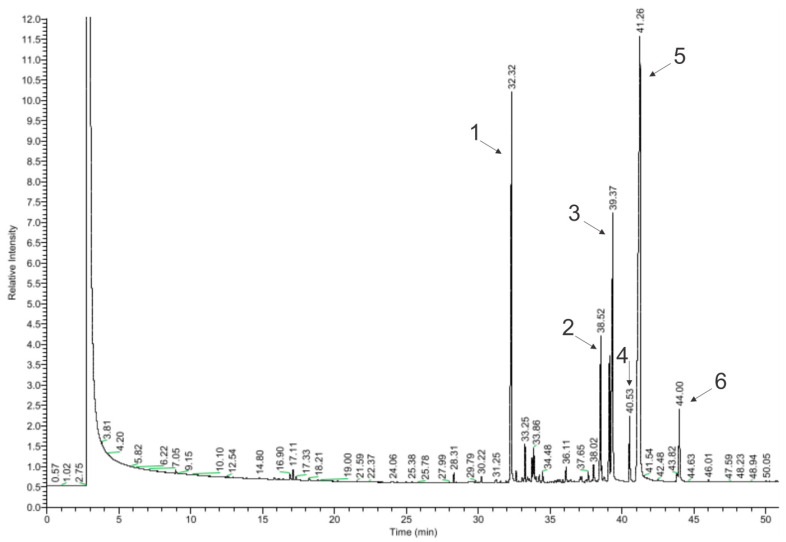
Chromatogram of chamomile essential oil (GC–MS); main compounds: 1—*β*-farnesene; 2—*α*-bisabolone oxide A; 3—*α*-bisabolol; 4—chamazulene; 5—*α*-bisabolol oxide A; and 6—cis-ene-yne-Dicycloether.

In total, 64 of the 99 detected chemical compounds were identified in chamomile essential oil (Figure 4, Table 4) and are consistent with the chromatographic profile provided by the manufacturer (Avicenna Oil, Poland) and with the literature data. Bisabolol oxide A was found in the largest amount (39.25%), which is within the specified value in the literature (2–60%), as well as in the chromatographic profile of the manufacturer and the European Pharmacopoeia (PE) (≥20%). The next largest amounts were *α*-farnesene (19.39%) (in the literature: approx. 8%) and *α*-bisabolol (14.87%) (in the literature: 1–60%; Avicenna Oil and PE: 10–65%) [17,18,20,28,29]. The compound that gives the oil its characteristic blue color, chamazulene, was found in an amount of 2.23%. This is consistent with the literature data, according to which this oil contains from 2 to 25% of chamazulene, while both the manufacturer’s specification and the European Pharmacopoeia state its content as equal to or above 1% [17,18,20,28].

In amounts above 1% in the EO, there were *α*-bisabolol B oxide (5.92%) (in the literature: 3–50%), trans-isopropilobicyclonon-3-en-8-one (4.26%), isomers 2-(2,4-hexadinilidene)-1,6-dioxa-spiro [4,4]non-3-ene (2.83% and 0.16%), *β*-farnesene (1.39%), and *β*-cubebene (1.23%), which are not provided in the literature as the main compounds contained in the oil. Among the effects reported in the literature as a consequence of this oil, the following were not detected: en-yn-dicycloether, n-octanal, 1,8-cineole, *α*-terpineol, germacrene D, and polyacetylene derivatives. Regarding the literature data, these compounds occur in chamomile oil in the following amounts: 8%, 6%, and 3.9%; and approx. 3%, 3%, and 4–18% [17,18,20].

According to the literature, *α*-bisabolol and chamazulene detected in chamomile essential oil have antimicrobial properties. Both of these compounds also have anti-inflammatory properties, and chamazulene also exhibits anti-allergic activity [21]. Accordingly, chamomile oil is a potentially biologically active substance and can be used to investigate its possibility to be released from the model insert.

The total amount of released compounds in the gas phase of cellulose agar media with chamomile essential oil is presented in Table 5. For each of the inserts with essential oil, the main compounds were identified and recorded after the specified incubation time. As the inserts were stored in the model systems, a reduction in the number of compounds in the headspace phase of the matrix was observed. Crystalline cellulose is not largely affected by the environmental moisture in comparison to, e.g., hemicellulose [30], which is beneficial to keep its performance in combination with other materials [31].

The detection of volatile compounds above the surface phase of BioTexPants with cellulose and microcrystalline cellulose was at a similar level. On the day of preparation, the inserts had from 15 to 19 compounds detected for the insert with cellulose and 14–19 compounds for the insert with microcrystalline cellulose. The most compounds were found for the OE:C and OE:CM ratios of 1:2. After 7 days of incubation, three to nine compounds were detected in the gas phase. After 14 days of storing the inserts, no volatile compounds of chamomile oil were detected in the matrix with the OE:C ratio of 1:1, one compound was detected above the insert with the 1:2 ratio, and six compounds were detected for the 1:3 concentration. For microcrystalline cellulose after 14 days of incubation there were eight, four, and three compounds, respectively. For the time intervals of 28 and 56 days, the results obtained were at a similar level for both celluloses. The largest number of compounds for these insert storage times was obtained for the OE:C 1:3 system (four compounds each).

In Table 6, the compounds identified in the gas phase above the surface of cellulose agar matrices with cellulose or microcrystalline cellulose and chamomile essential oil are listed.

On the day of preparing the inserts, in the gas phase, the following compounds were found above all matrices: artemisia ketone, *α*-farnesene, *β*-farnesene, *β*-caryophyllene, bicyclogermacrene, *δ*-cadinene, *α*-bisabolol, chamazulene, and *α*-bisabolone oxide. Of these compounds, bicyclogermacrene, *δ*-cadinene, and *α*-bisabolone oxide were not detected in chamomile essential oil because they were probably outside the detection and quantification range of the used method. The oil also did not contain *α*-caryophyllene (detected in EO:C 1:1 and 1:3, and EO:CM 1:1 and 1:2), 7-epi-1,2-dehydrosesquicineol, and *α*-amorphene (present in EO:C 1:2 and 1:3, and EO:CM 1:2). The main compound was *β*-farnesene, ranging from 64.62 to 68.58%. It was also one of the main chemical compounds in pure essential oil (19.39%).

In the gas phase above the surface of the insert, there were compounds which were not detected in chamomile essential oil: *α*-caryophyllene, 7-epi-dehydrosesquicineol, *α*-amorphene, germacrene D, bicyclogermacrene, *δ*-cadinene, *α*-bisabolone oxide A, and *α*-bisabolol A oxide. Germacrene D, bicyclogermacrene, *δ*-cadinene, *α*-bisabolone A oxide, and *α*-bisabolol A oxide are compounds mentioned in the literature as components of chamomile oil and occur in the following amounts: 3%, 0.64%, 12.81%, 2–60%, and 0.4–12%, respectively [17,18,20,32]. The content of *α*-caryophyllene (0.33%) was confirmed in the extract from live flowers obtained using the headspace method [17]. No information was found in the literature of the occurrence of 7-epi-dehydrosesquicineol and *α*-amorphene in chamomile essential oil.

After 7 days of storing the inserts, *β*-farnesene, *α*-bisabolone oxide A, and artemisia ketone were detected in the gas phase of the matrices. However, they were not identified on the day of the inserts’ preparation. The presence of germacrene D (absent in OE:CM 1:3) and *α*-bisabol (absent in EO:C 1:1 and EO:CM 1:3) was also found above most of the matrices. After the other storage periods (14, 28, and 56 days), *β*-farnesene and *α*-bisabolone oxide were the compounds most frequently identified in the gas phase.

## 4. Discussion

The multifunctionality of textiles in cosmetic, pharmaceutical, and fragrance aspects means that the materials do not only serve as clothing, but also serve other functions, e.g., improving health, skin condition, or well-being. Multifunctional textile products are used to produce, among others, cosmetic textiles, aromatherapy textiles, home textiles (curtains and pillows), sports textiles, and clothes [33]. The group of materials containing cosmetics, taking into account their impact on the body, includes products providing the following effects: slimming, moisturizing, energizing, perfuming, refreshing and relaxing, giving vitality, protecting against UV radiation, and improving firmness and skin elasticity [34,35].

The task of this type of product is to transfer health-promoting substances through their contact with human skin. Other groups include fabrics with fragrances or deodorizing substances [33]. Aromatherapy cosmetotextiles improve the comfort of the legs, e.g., by reducing their swelling. They may also have an energizing, relaxing effect and facilitate respiratory healing [36,37,38]. Popular products also include perfumed scarves, perfumed dresses, bras, moisturizing and energizing tights, as well as underwear with microcapsules of silk, designed to reduce cellulite and smooth the skin [39,40].

Essential oils and fragrances are widely used in the textile industry, mostly encapsulated in microcapsules. Release mechanisms of the microcapsule’s core for cosmetic textiles are friction, pressure, and biodegradation, and for aromatherapy and fragrance textiles, these methods are friction and diffusion through the polymer wall [36]. In the BioTexPants, the biological active compounds are immobilized on textiles thanks to cellulose agar film and they are released by higher temperatures, which is a brand-new solution. Thanks to that, compounds with anti-inflammatory and antibacterial activities may be released and support treatment of lower urinary tract inflammations.

The temperature of the treatment in BioTexPants is 40 °C, as it is close to the human body temperature and increases blood flow, which helps the treatment and supports the release of active compounds from an insert. It was found that temperature conditions are a significant factor influencing the concentrations of volatile organic compounds. Tests conducted by Stachowiak-Wencek and Pradzynski within the temperature range from 23 to 40 °C showed that an increase in temperature from 23 °C to 40 °C intensifies the release of volatile organic compounds. Moreover, it was found that temperature is an important factor determining the rate of volatile emission decay [41].

Changes in the chemical composition of the phase above the insert were observed during its storage, indicating a decreasing number of compounds probably caused by changes in the absorption of compounds by the cellulose agar film. In the pharmaceutical industry, to achieve good release characteristics, mixtures of various cellulose ethers or mixtures of different grades of a distinct polymer with different ratios can be used based on the intended release rate of the controlled release system [16]. In the BioTexPants, there is pure cellulose or microcrystalline cellulose bonded with agar film. There are no significant changes observed in the amount of compounds released while using cellulose or microcrystalline cellulose.

It is *α*-bisabolol which is mainly responsible for the antibacterial properties of chamomile essential oil. However, chamazulene also exhibits antimicrobial activity against Gram-positive bacteria [21]. *α*-Bisabolol and chamazulene were detected in the gas phase in an cellulose agar matrix with chamomile essential oil at a concentration of 1:1 on the day of the insert preparation (1.3% and 0.88%, respectively). In the EO:C 1:2 ratio, *α*-bisabolol was also identified after 7 days of storage (2.1% and 2.5%, respectively), while chamazulene was identified only on the day of preparing the inserts (0.7%). For the third ratio of 1:3, *α*-bisabolol appeared in the gas phase in all storage time intervals (1.7%, 1.8%, 2.1%, 2.5%, and 16%, respectively), while chamazulene only appeared on the day the inserts were prepared (1.6%).

In the gas phase of the matrix with microcrystalline cellulose in a 1:1 ratio to the EO, *α*-bisabolol was detected on the day of its preparation and after 7 and 14 days of storage, in amounts of 2.0%, 2.4%, and 1.5%, respectively. In the case of a matrix with an EO:MC ratio of 1:2, this alcohol was determined in the samples on the day of their preparation (1.9%) and after 7 (1.5%), 14 (2.1%), and 56 days (47.4%) of storage. For the EO:MC in the concentration of 1:3, *α*-bisabolol was identified on the day of preparation of the matrices (2.3%) and after 14 days of storage (19.1%). Chamazulene was identified for EO:MC in concentrations of 1:1 and 1:2 in the matrix only on the day of their preparation (0.5% and 0.3%, respectively).

The additional advantage of the designed BioTexPants solution is using non-woven fabric as an outer part of the insert, as non-woven fabrics are widely used as PPE in the medical sector. In the medical field, non-woven garments, wipes, and ancillary fabrics are attractive for their sufficient mechanical protection and excellent viral/bacterial barrier properties, while their disposable nature renders it easy to stay sanitary. Non-woven layers are highly absorbent, skin compatible, and air and moisture permeable [42].

Biotextronics is one of the rapidly growing fields, and the biosensor market is forecasted to reach 5 billion per year by 2025 [43]. The use of biotextronics systems is mainly based on monitoring the body’s vital signals (e.g., respiratory function, heart rate, body temperature, blood pressure, or neuromuscular response) and their dynamic changes [42,43]. Biotextronics systems supporting the diagnosis, therapy, and rehabilitation of patients are less often proposed [44]. The designed BioTexPants system is dedicated to patients suffering from recurrent urological infections, which fits into this narrow area of therapeutic biotextronics and supporting therapy. Our system is ultimately an attempt to modulate the microbiota of patients with the reduction or elimination of pathogenic microorganisms. The innovation of the system, apart from its usability, lies in the interaction between the active agent (essential oil) and pathogens of the lower urinary tract in order to achieve a therapeutic effect. To our knowledge, the presented research is one of the few applied to a textronics system to actively affect microorganisms colonizing the human body. There are solutions limited only to the use of a textronics sensor for *S. aureus* detection on human skin [45]. Due to the essential oil action in the gaseous phase, the assessment of the qualitative and quantitative composition of essential oil constituents in the suprasurface phase of the BioTexPants insert is crucial here.

According to the literature data, chamomile essential oil has antibacterial activity mainly due to the content of *α*-bisabolol and chamazulene; it can be concluded that the best effect in inhibiting bacterial growth should be demonstrated by the inserts on the day of their preparation, and to a lesser extent (due to the lack of chamazulene detection), inserts EO:C 1:2 and 1:3; EO:MC 1:1 and 1:2 after 7 days of storage; EO:C 1:3 and all variants with microcrystalline cellulose after 14 days of storage; and EO:C 1:3 after 28 and 56 days of storage. Since the micro-atmosphere above the surface of the insert immediately after the application of chamomile oil was composed of 14 to 19 oil constituents, regardless of the type of cellulose and the content of the oil, it is advisable to apply the oil to the surface of the insert directly before the use of the biotextronics system. Such a modification of the BioTexPants system would be recommended for the potential maximization of its antimicrobial effect. Moreover, “ready to use” inserts with chamomile oil with microcrystalline cellulose as a carrier or cellulose only in an oil-to-carrier ratio of 1:3 should be used up to 14 days after their manufacture. Due to significant changes in the chemical composition of the micro-atmosphere above the surface of the insert, the storage time of the inserts incorporating chamomile oil should not exceed 2 weeks.

The described research is at the fifth stage of technology readiness levels and requires further research. It is planned to investigate in situ the antimicrobial activity of the model inserts with chamomile EO against bacteria *Escherichia coli*, *Staphylococcus saprophyticus*, *Staphylococcus epidermidis*, *Enterococcus faecalis*, and *Pseudomonas aeruginosa*. After that stage, the authors will seek consent from the Medical Bioethics Committee to conduct research on humans, especially women.

## Figures and Tables

**Table 1:** Microorganisms causing lower urinary tract inflammation and their frequency of occurrence [6,7,8,9].

Microorganisms	Type and Frequency of Infections
*Escherichia coli*	80–85% non-hospital infections and 50% hospital infections
*Staphylococcus epidermidis*	5–10% infections
*Staphylococcus saprophyticus*	5–10% infections
*Pseudomonas aeruginosa*	mainly hospital infections
*Enterococcus faecalis*	mainly hospital infections
*Staphylococcus aureus*	mainly hospital infections
*Enterobacter* sp.	mainly hospital infections
*Klebsiella pneumoniae*	hospital infections, recurrent
*Proteus* sp.	recurrent infections
*Serratia* sp.	mainly hospital infections
*Mycobacterium* sp.	mainly hospital infections; blood-borne infections may occur
*Neisseria gonorrhoeae*	sexually transmitted bacteria
*Chalmydia trachomatis*	sexually transmitted bacteria
*Candida albicans, Cryptococcus neoformans, Aspergillus* sp.	blood-borne infection may occur

**Table 2:** Materials used in the outer insert preparation. All chemical compounds are 100% pure.

Compound	Producer	Target of Using
Non-woven viscose	Lentex S.A. (Lodz, Poland)	Outer insert layer for EO immobilization
Chamomile essential oil	Avicenna Oil^®^ (Wroclaw, Poland)	Antibacterial activity
Cellulose/Microcrystalline cellulose	RETTENMAIER Polska Sp. z o.o. (Warsaw, Poland)	Carrier for EO
Agar-agar	Sigma-Aldrich^®^ (Saint Louis, MO, USA)	Film for EO immobilization

**Table 3:** Characteristics of chamomile EO.

Organoleptic Description	Analytical Data	Chromatographic Profile
clear, viscous liquid, dark blue, with a characteristic odor	density (at 20 °C): 0.946–0.969 g/cm^3^ refractive index (at 20 °C): 1.496–1.516	(-)-*α*-bisabolol: 10–65% chamazulene: ≥1.0% bisabolol oxide and (-)-*α*-bisabolol: ≥20%

**Table 4:** Chemical composition of chamomile essential oil.

No.	Chemical Compounds	Content in EO [%]	Content According to Manufacturer’s Specifications Avicenna Oil [%]	Content According to European Pharmacopoeia 7 [%]
1	Ethyl 2-methylbutyrate	0.09	NA	NA
2	*α*-Pinene	0.01	NA	NA
3	Propyl 2-methylbutanoate	0.03	NA	NA
4	*β*-Fellandren	0.01	NA	NA
5	Yomogi alcohol	0.03	NA	NA
6	*β*-Cymene	0.05	NA	NA
7	*β*-Terpineol	0.02	NA	NA
8	*α*-Pinene	0.02	NA	NA
9	*β*-*cis*-Ocymene	0.20	NA	NA
10	Ketone artemisia	0.29	NA	NA
11	*γ*-Terpinene	0.09	NA	NA
12	Alcohol artemisia	0.05	NA	NA
13	Borneol	0.01	NA	NA
14	Eliksen	0.29	NA	NA
15	Decanoic acid	0.06	NA	NA
16	*α*-Kopaene	0.04	NA	NA
17	*β*-Elemen	0.20	NA	NA
18	*β*-Kubeben	0.06	NA	NA
19	*β*-Caryophyllene	0.08	NA	NA
20	Aromadendren	0.05	NA	NA
21	*β*-Farnesen	19.39	NA	NA
22	Junipen	0.31	NA	NA
23	*γ*-Muurolen	0.17	NA	NA
24	*β*-Kubeben	1.23	NA	NA
25	*α*-Farnesen	0.04	NA	NA
26	*β*-Selinen	0.13	NA	NA
27	Aromadendren	0.11	NA	NA
28	Eliksen	0.73	NA	NA
29	*α*-Farnesen	1.39	NA	NA
30	*α*-Bisabolen	0.13	NA	NA
31	*γ*-Kadinen	0.22	NA	NA
32	*β*-Kadinen	0.31	NA	NA
33	Sesquisabinene hydrate	0.04	NA	NA
34	*β*-Santanol	0.06	NA	NA
35	*β*-Caryophyllene oxide	0.03	NA	NA
36	Sesquisabinene 7-epi-*cis*-hydrate	0.08	NA	NA
37	Megastigmatrienon	0.08	NA	NA
38	Denderalasine	0.08	NA	NA
39	Spatulenol	0.50	NA	NA
40	Isoaromedendrene epoxide	0.03	NA	NA
41	*α*-Akorenol	0.10	NA	NA
42	Epiglobulol	0.05	NA	NA
43	Ledol	0.18	NA	NA
44	*β*-Santalol	0.14	NA	NA
45	Limonene-6-yl pivalonate	0.13	NA	NA
46	Bisabolol oxide A	0.37	≥20	≥20
47	Spatulenol	0.06	NA	NA
48	*τ*-Kandinol	0.68	NA	NA
49	Bisabolol oxide B	5.92	NA	NA
50	Cyclohexanecarboxylic acid	0.29	NA	NA
51	*α*-Himachalane	0.17	NA	NA
52	Trans-2-Isopropylbicyclo[4.3.0]non-3-en-8-one	4.26	NA	NA
53	*α*-Bisabolol	14.87	10–65	10–65
54	Chamazulene	2.23	≥1.0	≥1.0
55	Bisabolol oxide A	39.25	NA	NA
56	Hexahydrofarnesyl acetate	0.21	NA	NA
57	3-Piperidinopropyl-3-chlorobenzoate	0.16	NA	NA
58	*cis*-ene-yne-Dicycloether	2.83	NA	NA
59	Azulen-2-ol, 1,4-dimethyl-7-(1-methylethyl)-	0.06	NA	NA
60	Oleic acid	0.02	NA	NA
61	Linoleic acid	0.08	NA	NA
62	Ikozan	0.17	NA	NA
63	Heptakozan	0.37	NA	NA
64	Hydroquinone	0.05	NA	NA

**Table 5:** Quantitative composition of volatile compounds in the gas phase above cellulose agar matrices with chamomile EO: C—cellulose and MC—microcrystalline cellulose.

Time of Storage [Days]	Total Number of Identified Compounds					
	EO:C			EO:MC		
	1:1	1:2	1:3	1:1	1:2	1:3
0	15	19	15	14	19	16
7	7	9	9	5	9	3
14	0	1	6	8	4	3
28	0	2	4	2	2	1
56	0	2	4	2	2	1

**Table 6:** Summary of the composition of the gas phase of matrices with chamomile EO and cellulose or microcrystalline cellulose during storage and comparing it with the chemical composition of chamomile essential oil (blue font indicates compounds where their presence was found in all types of matrices).

No.	Chemical Compounds	EO:C			EO:MC		
		1:1	1:2	1:3	1:1	1:2	1:3
On the day of preparation							
1	Ethyl 2-methylbutyrate	ND ^1^	ND	ND	ND	ND	0.62
2	*α*-Pinene	ND	ND	ND	ND	ND	0.58
3	*β*-Ocymene	0.46	0.34	ND	ND	0.45	0.53
4	*β*-Ocymene	0.91	1.17	ND	ND	0.95	2.45
5	Artemisia ketone	1.05	1.04	0.46	0.80	0.97	2.54
6	*γ*-Terpinen	0.76	0.73	ND	ND	0.49	1.79
7	*β* -Farnesene	68.58	65.22	64.62	68.49	66.05	65.00
8	*α*-Caryophyllene	1.14	1.61	ND	1.00	1.23	ND
9	7-epi-1,2-Dehydrosesquicineol	ND	0.44	0.40	ND	0.61	ND
10	Aromadendren	1.00	0.95	2.41	0.96	1.09	ND
11	*α*-Amorphene	ND	0.81	0.99	ND	0.91	ND
12	*α* -Farnesene	7.42	6.99	7.68	7.42	7.17	5.84
13	*β*-Kubeben	ND	6.97	ND	ND	6.86	ND
14	Germacrene D	7.36	NA	7.68	7.42	ND	5.80
15	*β* -Caryophyllene	2.22	2.30	2.50	2.46	2.20	1.72
16	Bicyclogermacrene	3.69	3.72	4.13	3.78	3.33	3.04
17	*δ* -Kadinen	2.15	2.01	2.49	1.92	2.26	2.43
18	*α*-Bisabolol oxide B	ND	0.40	0.52	0.64	0.67	ND
19	*α* -Bisabolol	1.31	2.13	1.68	2.03	1.90	2.30
20	*α*-Bisabolone oxide A	1.06	1.52	1.34	1.41	1.51	1.41
21	Chamazulene	0.88	0.69	1.55	0.50	0.31	1.97
22	*α*-Bisabolol	ND	0.95	1.55	ND	ND	1.97
23	*α*-Bisabolol oxide A	ND	ND	ND	1.16	1.03	ND
After 7 days							
1	Artemisia ketone	1.13	0.90	0.31	1.48	0.53	2.71
2	*β* -Farnesene	84.70	72.03	80.13	87.26	78.70	90.85
3	Myrcene	ND	ND	5.03	ND	0.53	ND
4	*α*-Farnesene	ND	6.89	ND	ND	ND	ND
5	Aromadendren	ND	3.30	2.36	ND	2.31	ND
6	Germacrene D	4.58	6.89	5.03	4.63	5.33	ND
7	Selinen	2.86	ND	ND	ND	ND	ND
8	*β*-Caryophyllene	ND	ND	2.00	ND	ND	ND
9	Bicyclogermacrene	2.26	3.06	NA	ND	2.20	ND
10	*δ*-Kadinen	3.15	2.52	1.93	ND	2.53	ND
11	*α*-Bisabolol	ND	2.48	1.76	2.46	1.51	ND
12	*α*-Bisabolone oxide A	1.32	1.91	1.44	4.18	1.57	6.44
After 14 days							
1	Artemisia ketone	ND	ND	3.06	ND	2.01	ND
2	*β*-Farnesene	ND	100	82.05	83.10	92.42	68.68
3	*α*-Farnesene	ND	ND	5.04	3.84	ND	ND
4	*β*-Kubeben	ND	ND	5.04	ND	ND	ND
5	Germacrene D	ND	ND	ND	3.84	ND	ND
6	*β*-Caryophyllene	ND	ND	ND	1.94	ND	ND
7	Bicyclogermacrene	ND	ND	ND	1.60	ND	ND
8	*δ*-Kadinen	ND	ND	ND	1.82	ND	ND
9	*α*-Bisabolol	ND	ND	2.14	1.51	2.10	19.12
10	*α*-Bisabolone oxide A	ND	ND	2.67	2.35	3.46	12.19
After 28 days							
1	*β*-Farnesene	ND	69.83	88.57	70.15	65.46	ND
2	Germacrene D	ND	ND	4.65	ND	ND	ND
3	*α*-Bisabolol	ND	ND	2.46	ND	ND	ND
4	*α*-Bisabolone oxide A	ND	30.17	4.31	29.85	34.54	100
After 56 days							
1	*β*-Farnesene	ND	53.06	38.54	45.80	ND	ND
2	*α*-Bisabolol oxide B	ND	ND	11.62	ND	ND	ND
3	*α*-Bisabolol	ND	ND	16.04	ND	47.41	ND
4	*α*-Bisabolone oxide A	ND	46.94	33.80	54.20	52.59	100

^1^ ND—not detected.

## Data Availability

Samples of the essential oil and inserts are available from the authors.
